# Cathodoluminescence as a tracing technique for quartz precipitation in low velocity shear experiments

**DOI:** 10.1038/s41598-023-37052-w

**Published:** 2023-06-23

**Authors:** Maartje F. Hamers, André R. Niemeijer, Martyn R. Drury

**Affiliations:** Department of Earth Sciences, Utrecht University, Princetonlaan 8a, 3584 CB Utrecht, The Netherlands

**Keywords:** Structural geology, Geodynamics, Scanning electron microscopy

## Abstract

Two simulated gouges (a pure quartz and a quartz-muscovite mixture) were experimentally deformed in a ring shear apparatus at a constant low velocity under hydrothermal conditions favourable for dissolution–precipitation processes. Microstructural analysis using scanning electron microscope cathodoluminescence imaging and cathodoluminescence spectroscopy combined with chemical analysis showed that quartz dissolution and precipitation occurred in both experiments. The starting materials and deformation conditions were chosen so that dissolution–precipitation microstructures could be unambiguously identified from their cathodoluminescence signal. Precipitated quartz was observed as blue luminescent fracture fills and overgrowths with increased Al content relative to the original quartz. In the pure quartz gouge, most of the shear deformation was localized on a boundary-parallel slip surface. Sealing of fractures in a pulverized zone directly adjacent to the slip surface may have helped keeping the deformation localized. In the quartz-muscovite mixture, some evidence was observed of shear-accommodating precipitation of quartz in strain shadows, but predominantly in fractures, elongating the original grains. Precipitation of quartz in fractures implies that the length scale of diffusive mass transfer in frictional-viscous flow is shorter than the length of the quartz domains. Additionally, fracturing might play a more important role than generally assumed. Our results show that cathodoluminescence, especially combined with chemical analysis, is a powerful tool in microstructural analyses of experimentally deformed quartz-bearing material and visualizing quartz precipitation.

## Introduction

Fluid-rock interactions, such as dissolution and precipitation processes, play an important role in both natural and experimental deformation and faulting. Dissolution–precipitation creep (also known as pressure solution) is widely accepted as a relevant deformation and fault healing mechanism in shear settings in nature and experiments^1^. Usually the occurrence of dissolution–precipitation is inferred from signs of dissolution, such as the presence of grain truncations and indentations, irregular grain boundaries, porosity reduction, and from healed fractures that can sometimes be recognized as traces of fluid inclusions^2–5^. Furthermore, euhedral quartz grains observed in high temperature shear experiments on quartz gouge have been interpreted as evidence for precipitation^5^. The presence of newly precipitated material is, however, not routinely demonstrated, and in most microstructural studies it is difficult or impossible to distinguish overgrowths and healed fractures from the original grains. However, knowing where material precipitates is very relevant, as this provides information on the role of solution transfer processes in the deformation (e.g., deformation-accommodating or only facilitating compaction) and on the length scales involved in diffusive mass transfer, which control the overall deformation rate produced by dissolution and precipitation.

Cathodoluminescence (CL) can reveal subtle variations in chemistry and structure of minerals^6^ and both CL imaging and hyperspectral analysis have been a powerful tool in the study of quartz for decades^7–16^. Giger et al.^17^ used CL imaging to show that dissolution and precipitation of quartz occurred in hot pressing experiments: greyscale SEM-CL images and quantitative chemical mapping revealed a slightly increased Al content in quartz overgrowths in a natural pure quartz powder sample hot pressed for eight hours at 850 °C under a confining pressure of 250 MPa, pore fluid pressure of 150 MPa and differential stress of 90 MPa. Williams et al.^18^ used a cold-cathode CL system on a petrographic microscope to trace quartz precipitation in healed fractures and overgrowths after long duration quartz growth experiments (at 300–450 °C and 150 MPa, with an Al-enriched pore fluid containing amorphous silica). Recently, various authors used CL to visualize recrystallized quartz in experimentally and naturally deformed rocks, linking CL to trace element re-equilibration (especially Ti) resulting from the presence of grain boundary fluids during deformation, both at low and high temperatures^19–22^.

In this study, we demonstrate the use of CL to reveal quartz precipitation during low velocity shear experiments on a pure quartz gouge and a mixed quartz and muscovite gouge. We used carefully selected and characterised starting materials so that dissolution–precipitation microstructures could be unambiguously identified from their cathodoluminescence signal. Our results show that both SEM-CL images and CL hyperspectral maps are effective tools in microstructural studies of experimentally deformed quartz, that clearly highlight newly precipitated quartz in overgrowths and healed fractures. Furthermore, our results indicate that the length scale of dissolution and precipitation is related to the spacing of sealed fractures in relict quartz grains.

## Material and methods

### Starting material and experimental methods

Material from two shear experiments was considered for this study: a pure quartz gouge (experiment u742) and an 80 wt% quartz–20 wt% muscovite mixture (experiment u195) that was previously described by Niemeijer^4^. The starting material for u742 was a single crystal of quartz showing relatively homogeneous CL emission (mostly red with some growth zoning revealed by blueish to violet zones). The initial CL characteristics of the starting material allowed any quartz precipitation (with different CL behaviour due to chemistry and/or defect structure resulting from the experimental conditions and setup) to be readily identified. The crystal was crushed manually and sieved to a grainsize of < 63 µm to obtain a simulated gouge for shear experiments. The gouge was compacted at 500 °C and effective normal stress σ_n_^eff^ = 120 MPa for 56 h before starting shearing. A reference sample was not sheared, but only compacted at room temperature for 30 min. Experiment u195 (described previously in Niemeijer^4^) was done on a mixture of 80 wt% quartz (Sil-co-sil 49, U.S. Silica company) and 20 wt% muscovite (high purity muscovite single crystals, grade V4, SPI supplies, crushed and sieved at d < 50 µm). The quartz in this material has grains with different origins and therefore a variety of initial CL colours and microstructures.

The shear experiments were conducted using a hydrothermal ring shear apparatus described previously by e.g. Niemeijer et al.^3^. Conditions were chosen suitable for the occurrence of dissolution and precipitation in quartz. Experiment u742 was conducted at a temperature of T = 500 °C for the first part of the experiment, which was then increased to 600 °C because the sample showed stick–slip behaviour; effective normal stress σ_n_^eff^ of 120 MPa, fluid pressure P_f_ of 100 MPa and a constant shear velocity v of 0.03 µm/s, with a total load-point displacement of 14.294 mm. Experimental conditions for u195 were similar, at a temperature T of 500 °C, effective normal stress σ_n_^eff^ of 120 MPa, fluid pressure P_f_ of 80 MPa and a constant shear velocity v of 0.03 µm/s, with a total displacement of 30.9 mm. After the experiments, shear stress was removed, the samples cooled within ~ 30 min and fluid pressure and normal stress were removed. Bulk strain and strain rates were 12.1 and 2.65e−5 for the quartz gouge experiment, and 38.3 and 3.72e−5 for the quartz-muscovite gouge experiment. After recovering samples from the ring shear apparatus, shear sense (top-to-the-left or -right) is unclear, but figure captions indicate whether shear direction is in the image plane horizontal or in the viewing direction.

### Sample preparation and microanalysis

Fragments of experimental material were embedded in epoxy, then ground down and polished using Al_2_O_3_ down to a grainsize of 0.3 µm, after which a final polishing step was done using colloidal silica (Syton) to remove surface damage induced by mechanical polishing. Due to charging problems resulting from differential polishing of quartz and muscovite, sample u195 was also polished in a broad ion beam polisher (Fischione Model 1061 SEM Mill) for three hours with a 2 kV beam at an angle of 1° with 0% focus. Samples were coated with a thin layer of carbon to prevent charging in the electron microscope.

Backscattered electron (BSE), secondary electron (SE) and colour filtered cathodoluminescence (CL) images were recorded using an FEI Helios Nanolab G3 scanning electron microscope with Gatan PanaCL detector (detection range 185–850 nm) including red (595–850 nm), green (495–575 nm) and blue (185–510 nm) filters. Red, green, and blue filtered SEM-CL images were optimized for brightness and contrast (levels) using Adobe Photoshop, and in some cases a noise reduction filter was applied. The filtered images were then combined by including each greyscale image into the red, green, and blue channel respectively of a new RGB colour image. Additional false-colour SEM-CL images were acquired on a Zeiss Gemini 450 with Delmic SPARC Compact CL system, by combining three filtered images (625–675 nm, 525–575 nm and 425–475 nm) into an RGB image using the RGB image plugin in the CL control software (Odemis). Beam settings on both instruments were typically 10 kV and 3.2–6.4 nA with a dwell time of 10–30 µs per pixel.

Electron backscattered diffraction (EBSD) maps were recorded using an FEI Helios Nanolab G3 or Philips XL30 SFEG scanning electron microscope, both with a Nordlys camera and Oxford Aztec 3.4 software, or a Zeiss Gemini 450 with Oxford Symmetry detector and Aztec 4.4. Beam settings for EBSD mapping were 20–30 kV and 2.5–90 nA with a 0.3–0.5 µm step size, at 70° sample tilt. EBSD data processing was done in Aztec Crystal 2.0 by first removing wild spikes, followed by iterative (max. 10 times) extrapolation of 8, 7 and 6 nearest neighbours. To plot pole figures of one orientation per grain, Dauphiné twins were removed by removing pseudosymmetry (60° rotation around <0001>).

CL hyperspectral maps and qualitative wavelength dispersive spectrometry (WDS) maps for Al and Ti were recorded using a JEOL JXA-8530F Hyperprobe field emission electron probe microanalyser with xCLent IV CL spectrometry system (detection range 197–975 nm) using a 200 µm aperture. Beam settings were 7–15 kV and 20–100 nA with dwell times of 20–500 ms and step sizes of 0.2–0.5 µm. CL spectra were analysed by fitting Gaussian peaks to the spectra using Fytik^23^.

## Results

### Mechanical data

**Figure 1:**
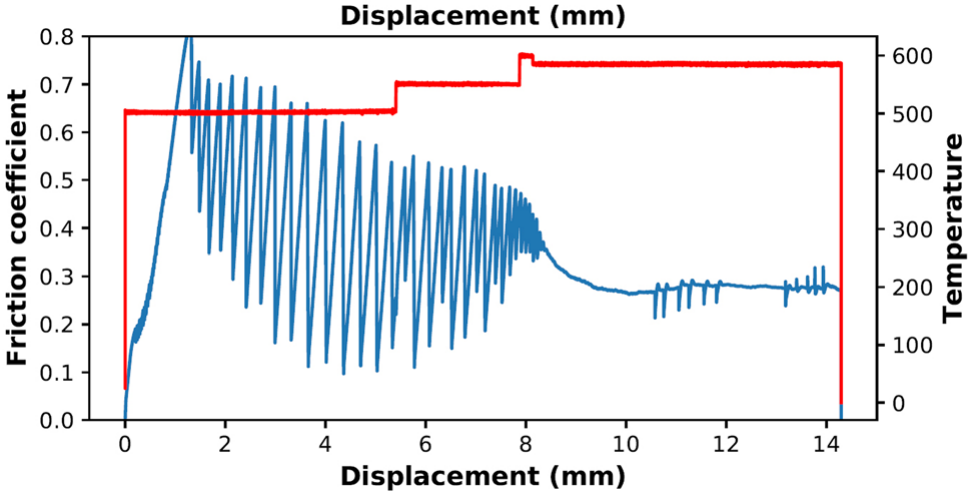
Mechanical data for the quartz gouge experiment (u742): displacement against friction coefficient and temperature.

Figure 1 shows the friction coefficient (shear stress divided by effective normal stress, ignoring cohesion) and temperature during the experiment as a function of displacement for the pure quartz experiment (u742). Friction increases quasi-linearly until failure occurs at a peak friction of ~ 0.85, followed by periodic stick–slip events with a decrease in both the peak and valley values of friction, resulting in a steady friction drop of ~ 0.4–0.5. Temperature was increased to 550 °C in an attempt to stabilize sliding and activate pressure solution-accommodated deformation^24^. The temperature increase resulted in slightly smaller stick-slips, but not stable sliding, and temperature was increased further to 600 °C. This resulted in stable sliding and considerable strain weakening until a friction level of 0.27. Subsequent steps in effective normal stress (between ~ 10 and 12 mm displacement) did not affect the level of friction, indicating a linear dependence of shear stress on effective normal stress. Internal friction, as established from the slope, at this stage of the experiment was 0.28. Layer thickness evolution indicates that compaction mainly took place in the second half of the experiments, after the temperature was raised from 500 to 600 °C.

The friction coefficient during the experiment on the quartz-muscovite mixture (u195) increased to 0.64 at the start of shearing, followed by weakening and stable sliding at a final friction coefficient of 0.32 (see Niemeijer^4^ for a more detailed description of this experiment).

### Microstructures

#### Quartz gouge

**Figure 2:**
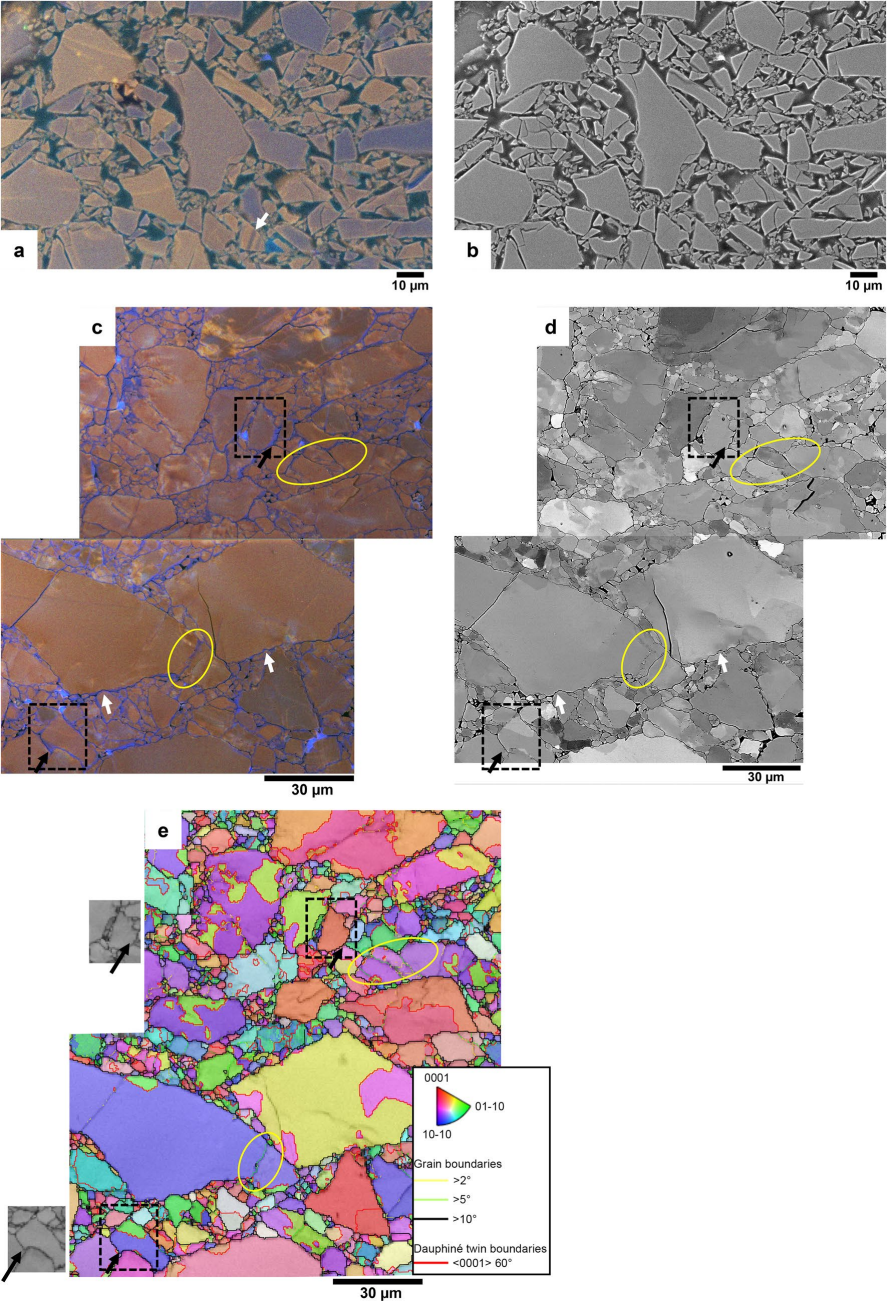
(**a**) False-colour RGB SEM-CL image of the crushed and compacted, but unsheared quartz starting material. White arrow indicates red and violet zoning pattern present in the original crystal. (**b**) SE image corresponding to the CL image in (**a**). (**c**) False-colour RGB SEM-CL image overlay on SE image, showing the microstructure in the main body of the sheared quartz gouge (u742). Shear direction is perpendicular to the image plane. Blue quartz seems to indent into red quartz in several locations (white arrows). Yellow ellipses show (partially) sealed fractures indicated as low-angle boundaries in the EBSD map in (**e**). (**d**) BSE image corresponding to a (also showing orientation contrast due to electron channelling effects). (**e**) EBSD IPF x map overlay on band contrast map, with grain boundaries (> 10° misorientation) in black, Dauphiné twin boundaries (60° rotation around the c-axis) in red, low-angle boundaries (5°–10°) in green and very low-angle boundaries (2°–5°) in yellow. Yellow ellipses show low-angle boundaries that can be recognized as (partially) blue luminescent sealed fractures in the SEM-CL image in c. Greyscale insets show EBSD band contrast (pattern quality) maps of the areas indicated with dashed black boxes. Pattern quality is the same in blue and red luminescent quartz (clearly blue luminescent areas indicated with black arrows, also shown in the CL image in c). Shear direction in c, d, and e is in the viewing direction (perpendicular to the image plane).

**Figure 3:**
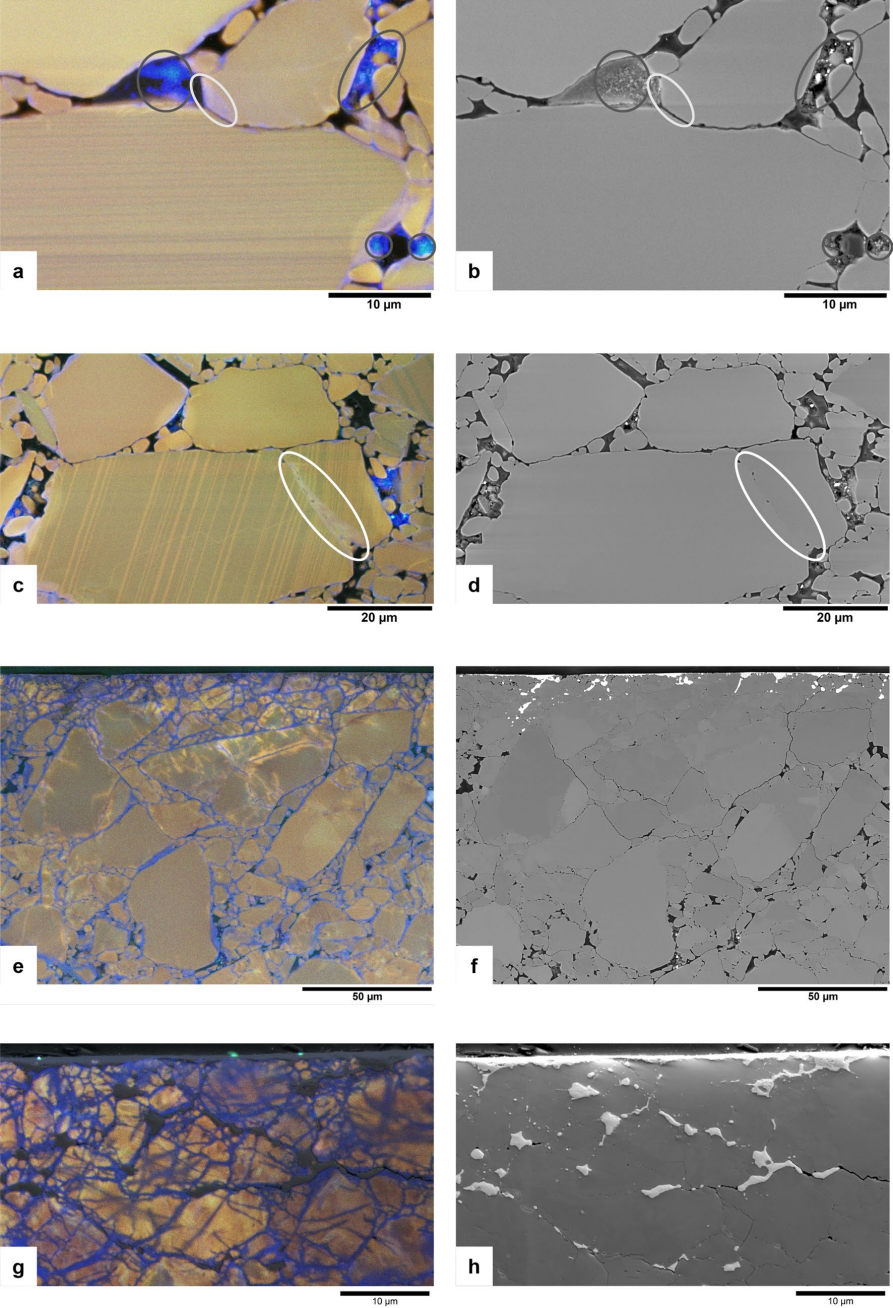
Microstructure in the sheared quartz gouge (u742). Shear direction is in the image horizontal (probably top to the left), except in g and h. (**a**) False-colour SEM-CL image overlay on BSE image showing indentation of a grain with a blue luminescent part into a grain with fine growth zoning (light grey ellipse). For comparison, blue luminescence from polishing grains in a pore is indicated by dark grey ellipses. The dark and light grey ellipses are also shown on the corresponding BSE image in (**b**), to confirm the different appearance of quartz showing variations in CL emission and pore space or remnant polishing material (which is present in pores and shows strong bright blue CL). (**c**) False-colour RGB SEM-CL image overlay on BSE image showing a fracture (white ellipse) in a finely zoned quartz fragment, sealed with pinkish blue luminescent quartz. The fracture is wider on the bottom right, suggesting opening from that direction. (**d**) BSE image corresponding to c, with white ellipse in the same location as in c, to confirm the presence of quartz in the pinkish sealed fracture. (**e**) False-colour RGB SEM-CL image overlay on BSE image of the pulverized zone next to the slip surface. Fine-grained red luminescent quartz fragments are sealed together by blue luminescent quartz in fractures, pore space and grain rims. Brighter red to yellow luminescent patches are present near the slip surface. (**f**) BSE image corresponding to the CL image in e. Ni-rich material, injected from the slip surface into the gouge, is clearly recognized by the high brightness (dark in CL). (**g**) False-colour RGB SEM-CL image overlay on SE image of the pulverized zone next to the slip surface (slip direction for this image is in the viewing direction), showing blue luminescent quartz in fractures and grain rims, brighter red to yellow luminescent patches near the slip surface and injected metal near the slip surface (dark in CL; bright white in SE in **h**). (**h**) SE image corresponding to the CL image in (**g**).

**Figure 4:**
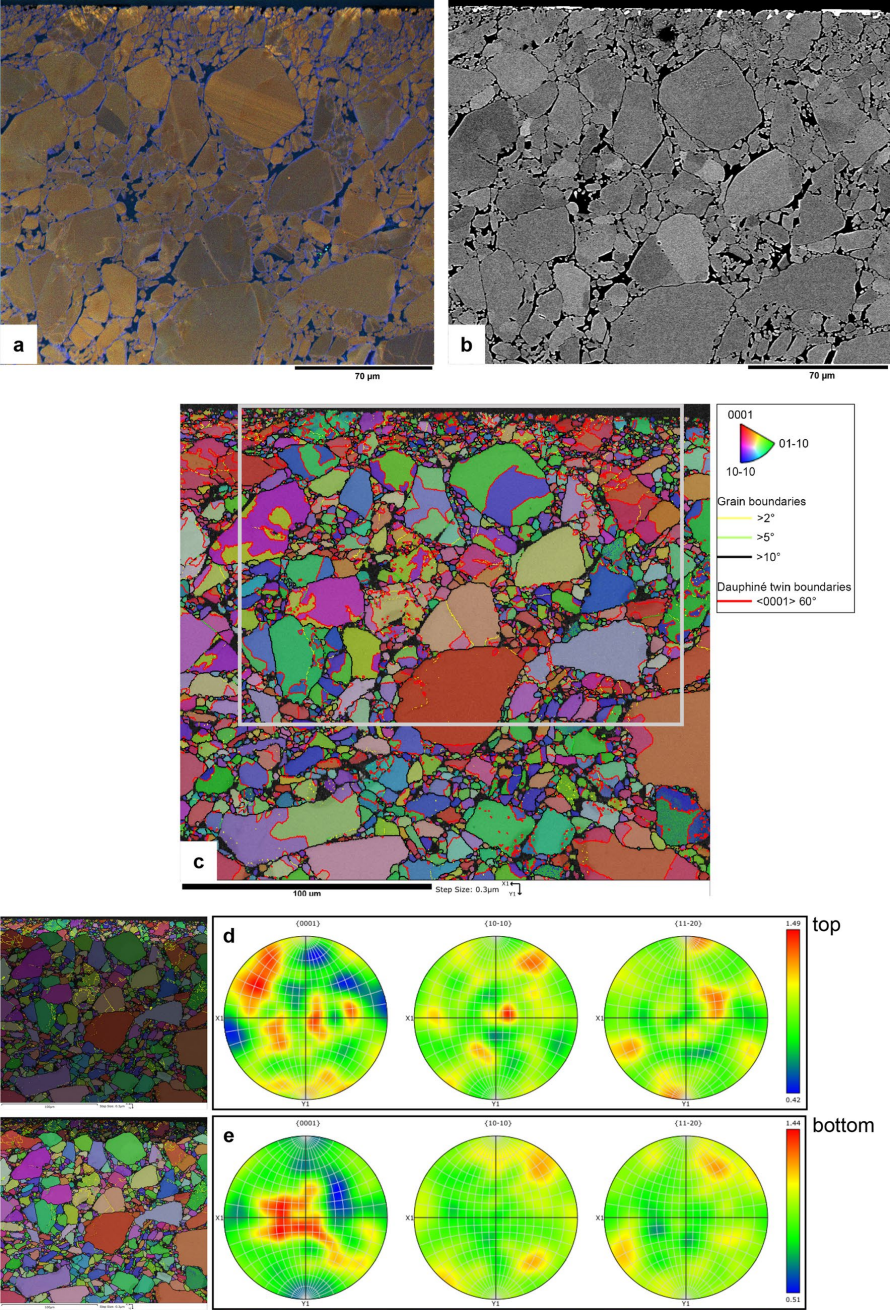
(**a**) False-colour RGB SEM-CL image and (**b**) corresponding BSE image. Shear direction is in the image horizontal (probably top to the left). (**c**) EBSD IPF x map overlay on band contrast map, with grain boundaries (> 10° misorientation) in black, Dauphiné twin boundaries (60° rotation around the c-axis) in red, low-angle boundaries (5°–10°) in green and very low-angle boundaries (2°–5°) in yellow. Grey box shows the location of the CL and BSE images in (**a**) and (**b**). (**d**) Orientation density plot (equal area, lower hemisphere) for the top fine-grained part of the map in (**c**). (**e**) Orientation density plot (equal area, lower hemisphere) for the rest of the map in (**c**). Both top and bottom slow a low MUD (multiples of uniform distribution) of ~ 1.5, indicating no significant difference in crystal preferred orientation between the top (near the slip surface) and the rest of the sample. X1 and Y1 in the pole figures in d and e refer to X1 and Y1 axes of the EBSD map in (**c**), and are in the sample reference system.

The grains in the crushed and compacted, but unsheared, quartz gouge reference sample (Fig. 2a) show the same homogeneous, mostly red to violet CL emission in SEM-CL images as the uncrushed crystal. Fragments are angular and some fragments with growth bands and zoning can be recognized (white arrow in Fig. 2a). In u742 (Figs. 2c,d, 3 and 4), sheared at 0.03 µm/s, porosity has almost disappeared in parts of the sample, notably near the slip surface, and grain boundaries are irregular, showing indentations and overgrowths. Thin blue luminescent rims and overgrowths (typically 0.5–2 µm wide) are observed on most grains throughout the sample. Sealed fractures show the same blue luminescence and occur mainly in larger grains. This blue luminescent quartz is not observed in the compacted, unsheared starting material. In several grains in u742, blue luminescent rims or overgrowths seem to indent into red luminescent grains (white arrows in Fig. 2c). The false-colour RGB SEM-CL image in Fig. 3a shows indentation of a grain with a blueish luminescent part into a grain with fine growth zoning (white arrow). A pinkish blue overgrowth on the zoned grain is indicated by a black arrow. Some sealed fractures are wider on one side, suggesting they opened from that direction (e.g., white ellipse in Fig. 3c).

CL images and EBSD maps (Figs. 2c–e and 4) show significant changes in grain shape after deformation, compared to the unsheared starting material shown in Fig. 2a. The larger grains remain angular to sub-angular, while the smaller grains are rounded or equi-axed to polygonal in shape. Many relict grains contain a few low-angle boundaries (2°–10° misorientation), which correspond with (partially) sealed fractures in the CL image. Examples are indicated with yellow ellipses in Fig. 2c–e. Figures 2c–e and 4 show that rounded to equi-axed grains are produced, with pores bounded by smoothly curved or straight interfaces. Grain boundaries show signs of smaller scale pores along the boundaries (e.g., the boundary to the right of the light grey ellipse in Fig. 3a,b).

**Figure 5:**
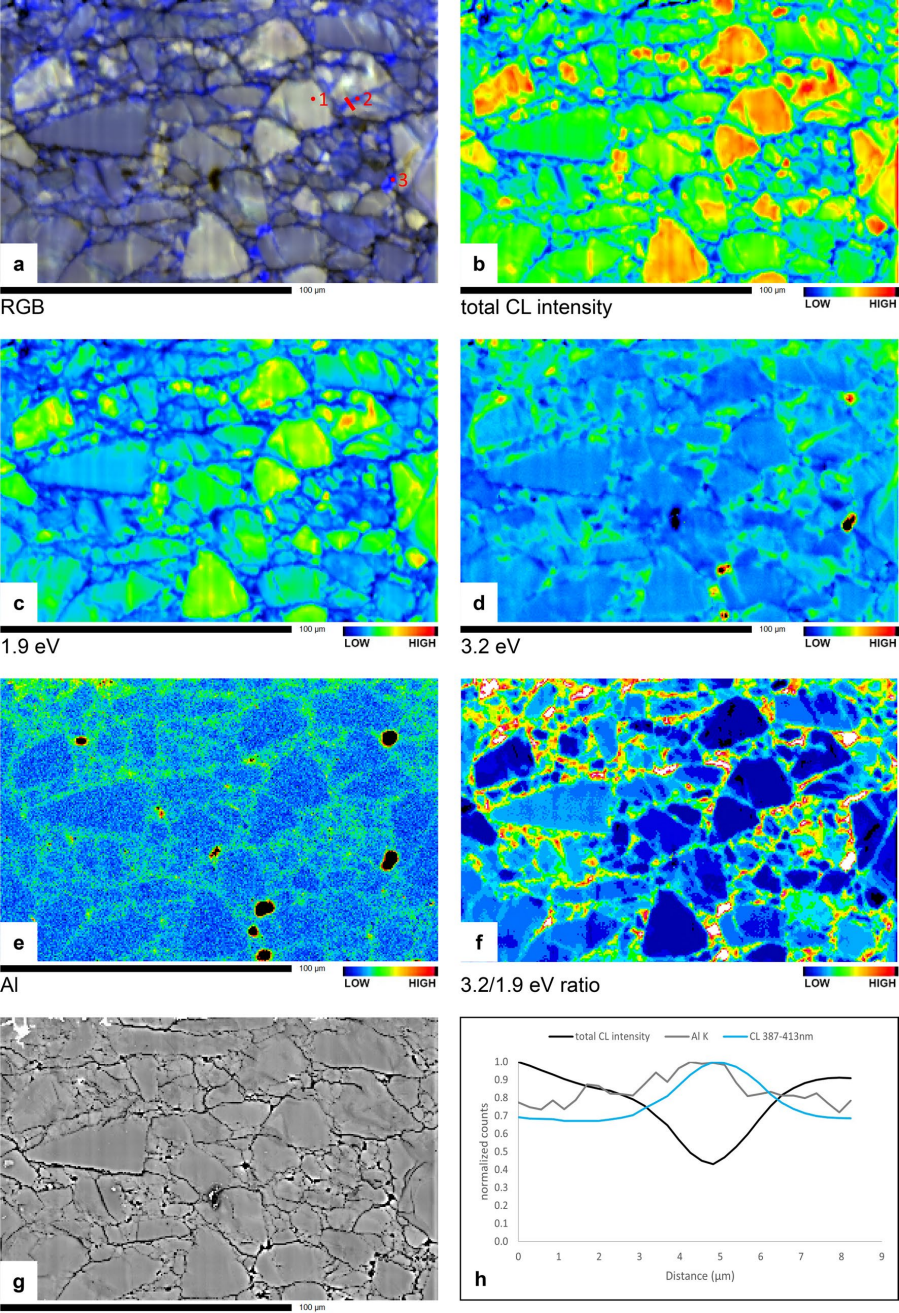
Microprobe maps of the sheared quartz gouge sample (u742), showing a correlation between blue CL and increased Al concentration. Shear direction is in the viewing direction. (**a**) False-colour RGB hyperspectral CL map. CL spectra of points 1–3 are shown in Fig. 6a, b and d respectively, the profile is shown in Fig. 5h; (**b**) total CL intensity; (**c**) 1.9 eV (650 nm; red) CL emission intensity; (**d**) 3.2 eV (390 nm; blue) CL emission intensity; (**e**) qualitative Al concentration; (**f**) 3.2/1.9 eV CL emission intensity ratio; (**g**) BSE (**h**) Profile line across the blue sealed fracture in 5a (left of point 2) showing normalized counts of total CL intensity (black line), 3.2 eV CL intensity (blue line) and Al concentration (grey line).

Ti content in u742 was below detection limit, but Al WDS maps show variation in Al concentration. Figure 5 shows a spatial correlation between blue CL emission (Fig. 5a,d,f) and increased Al content (Fig. 5e). This is further illustrated by the profile in Fig. 5h showing normalized counts of total CL intensity, 3.2 eV/400 nm (blue) CL intensity and Al.

**Figure 6:**
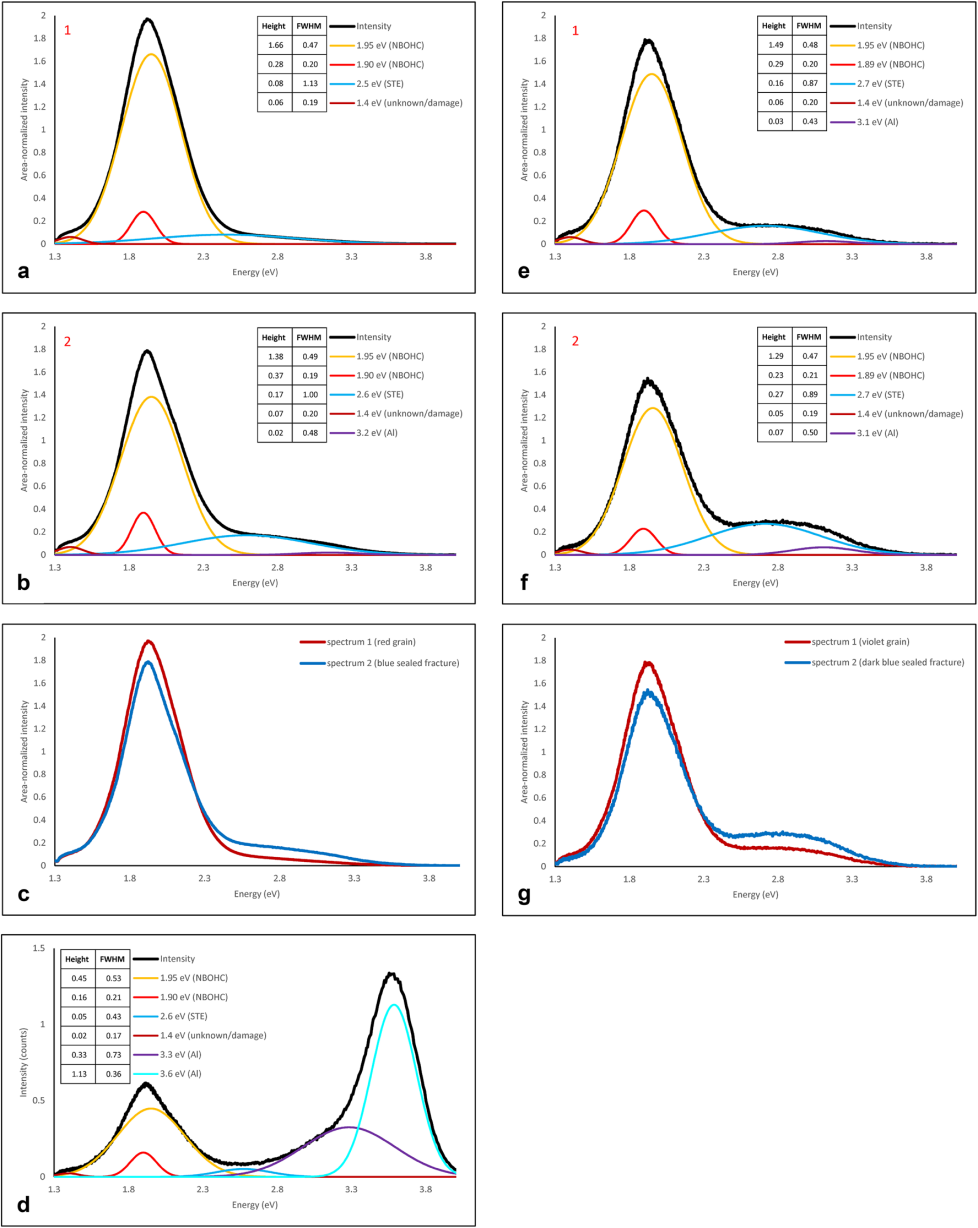
(**a**) Peaks fitted to the CL spectrum of the reddish to violet luminescent original quartz in point 1 in Fig. 5a. (**b**) Peaks fitted to the CL spectrum of blue quartz in the sealed fracture in point 2 in Fig. 5a. (**c**) Direct comparison of the CL spectra from point 1 and point 2 in Fig. 5a. (**d**) Peaks fitted to a CL spectrum from polishing powder (point 3 in Fig. 5a), showing a strong 3.6 eV peak related to Al_2_O_3_ and weaker contribution of quartz also sampled by the electron beam (i.e. the 1.9, 1.95 and 2.6 eV peaks also recognized in Fig. 6a,b,e,f). **e)** Peaks fitted to the CL spectrum of the reddish to violet luminescent original quartz in point 1 in Fig. 8a. (**f**) Peaks fitted to the CL spectrum of blue quartz in the sealed fracture in point 2 in Fig. 8a. (**g**) Direct comparison of the CL spectra from point 1 and point 2 in Fig. 8a. Abbreviations used in legends: Height = highest intensity of fitted peak; FWHM = full width at half maximum; NBOHC = non-bridging oxygen hole centre; STE = self-trapped exciton.

The CL emission from blue rims and healed fractures is characterized by a lower total intensity (Fig. 5b), a lower red emission (Fig. 5c) and a slightly higher blue emission with respect to the red to purple original grains (Fig. 5d,f). Peak fitting to CL spectra (Fig. 6a–c) shows that for both the red to violet quartz grains and for the blue quartz in rims and healed fractures, the main peak in the emission spectra is in the red range around 1.9 eV (650 nm), with a weaker broad blue peak around 2.7–2.8 eV (450–460 nm) (varying between 2.4 and 2.8 eV, probably because such a broad relatively weak peak is hard for the software to place precisely). The blue peak is relatively slightly stronger in the blue luminescent quartz than in the red to violet quartz (see normalized spectra plotted together in Fig. 6c). Furthermore, the best fit for spectra from the blue luminescent areas (Fig. 6b) contains a small extra peak at 3.1–3.2 eV (380–390 nm) that is not present in the best fit for the red to violet original quartz (Fig. 6a). The blue/red CL emission ratio, expressed as the 3.2/1.9 eV ratio, correlates well visually with the relative Al concentration (compare Fig. 5e,f).

On one side of sample u742 the shape of the piston teeth is clearly recognizable, but on the other side piston teeth are not present and a shiny surface developed, which is interpreted as a boundary-parallel slip surface. In reflected light microscopy the slip surface has a dark appearance, and backscattered electron (BSE) images and energy dispersive x-ray spectroscopy (EDS) analyses show the presence of mainly metal components (Ni, S, and some Fe, Ti, Cr, Co, and Al) on the surface. Some of these, notably Ni and S, have been injected into the sample to ~ 30 µm depth (CL-dark/SE-bright material in Fig. 3e–h, and top parts of Fig. 4a,b). A pulverized zone of a few tens of µm thickness with many sealed fractures and fine grains is observed near the slip surface (Figs. 3e,g and 4a). More blue luminescent quartz is present in the pulverized zone and closer to the slip surface than deeper into the sample. The surface area of blue luminescent quartz in SEM-CL images varies between ~ 2% in the body of the sample to ~ 23% in the pulverized zone directly adjacent to the slip surface. Furthermore, porosity is reduced in this zone (~ 2% of image surface) relative to deeper into the body of the sample (~ 6% of image surface). Patches of brighter red to yellow CL emission are present in some quartz grains, especially next to the slip surface. Some yellow CL patches cover almost an entire grain (Fig. 3e,g and 4a), but in some cases only small areas near fractures or crack tips are observed (see Figs. 2c and 3e). CL emission spectra from these yellow areas show an increased intensity, in particular of the 1.9 eV (650 nm) peak.

EBSD data (example map shown in Fig. 4) show a slight grain size reduction in the fractured zone close to the slip surface with respect to the body of the sample (in three different maps, average grain diameter in the top ~ 20–30 µm varies between 1.5 and 2.3 µm, versus 2.3–3.5 µm in the body of the sample). Crystallographic preferred orientation (CPO) is weak to not significant (MUD, multiples of uniform density, of ~ 1.5) both close to the slip surface and in the body of the sample (Fig. 4d,e). Furthermore, the blue luminescent quartz in rims and healed fractures shows the same diffraction pattern quality as the original (red to violet luminescent) quartz grains (see black arrows in Fig. 2c–e), indicating that the degree of crystallinity is similar in original and precipitated quartz. Comparison of SEM-CL images and EBSD maps of the same sample area (Fig. 2c,e and 4) shows that some sealed fractures (blue in CL) form grain boundaries (misorientation > 10°, indicated by black lines in EBSD maps), while others correspond to low angle boundaries (misorientation < 10°, indicated by green or yellow lines in EBSD maps).

### Quartz-muscovite gouge

**Figure 7:**
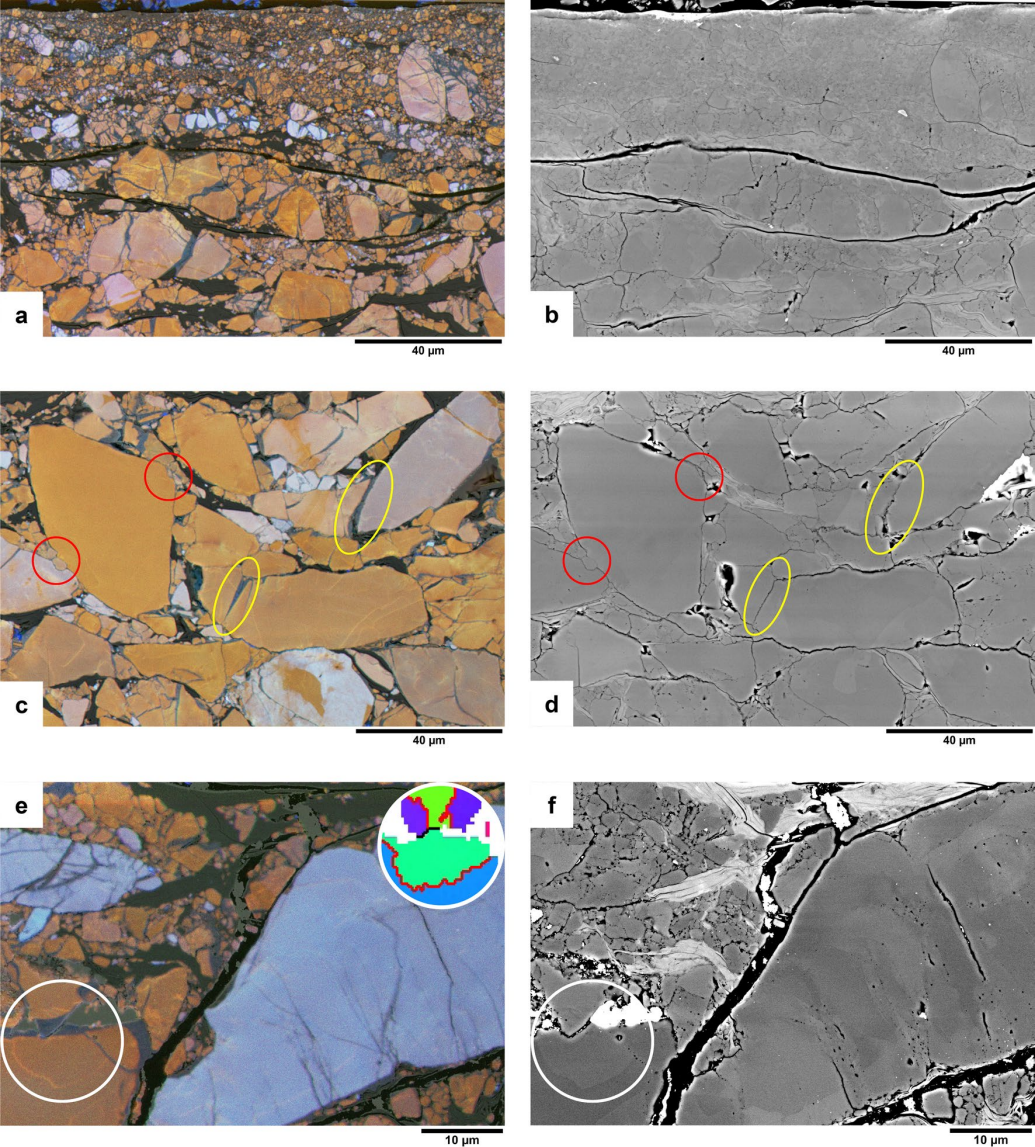
Dissolution effects (red circles) and dark blue CL overgrowths, pore fills and healed fractures (yellow circles) in the mixed quartz-muscovite gouge (u195). Shear direction is in the image horizontal (probably top to the left). False-colour RGB SEM-CL overlays on BSE images (**a, c**) and corresponding BSE images (**b, d**). (**e**) False-colour RGB SEM-CL image overlay on BSE image showing a dark blue luminescent overgrowth indenting into a red luminescent grain, with a brighter red line in a half-circle below the indentation point (white circle). EBSD measurements (see inset in top right white circle, same legend as Figs. 2e and 4c) indicate this is a Dauphiné twin boundary. (**f**) BSE image of the same are as shown in e (bright white areas are remains of a previous platinum coating).

**Figure 8:**
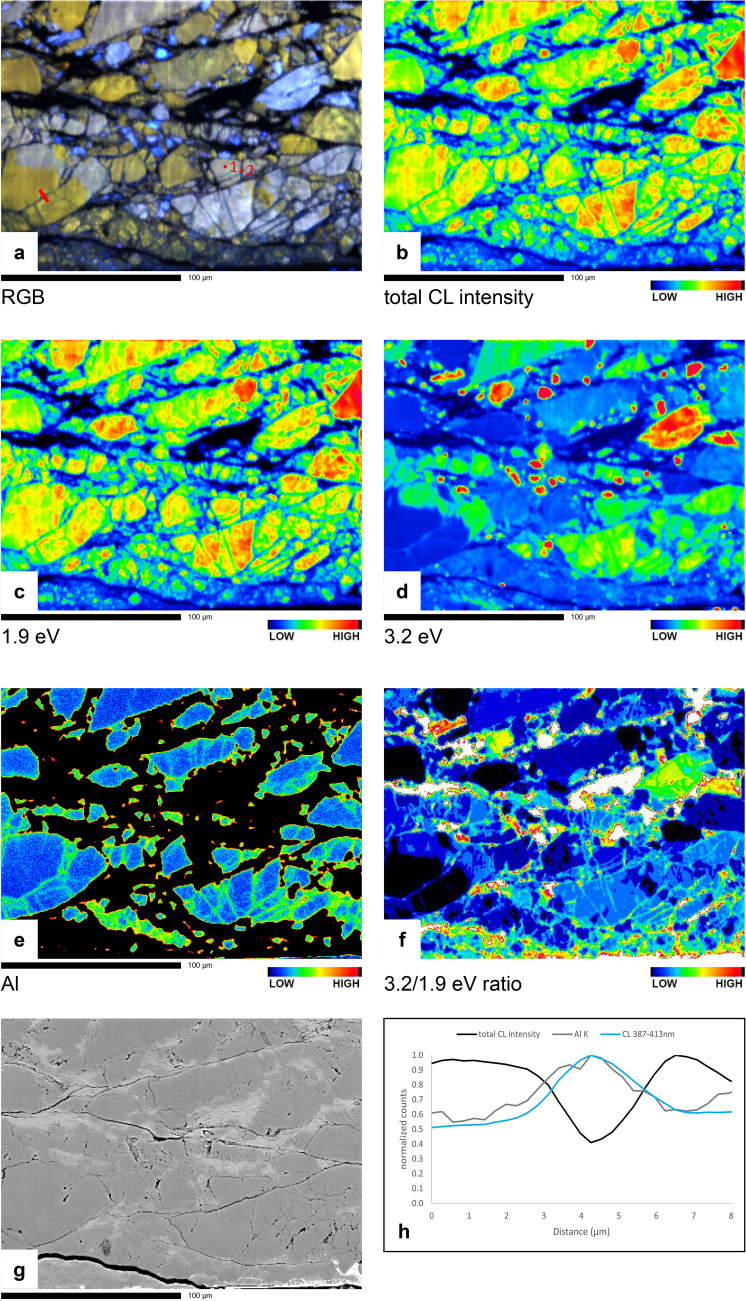
Microprobe maps of the sheared quartz-muscovite gouge sample (u195). Shear direction is in the image horizontal (probably top to the left). (**a**) False-colour RGB hyperspectral CL map; (**b**) total CL intensity; (**c**) 1.9 eV (650 nm, red) CL emission intensity; (**d**) 3.2 eV (390 nm, blue) CL emission intensity; (**e**) qualitative Al concentration; (**f**) 3.2/1.9 eV CL emission intensity ratio; (**g**) BSE (SEM image). (**h**) Profile line across the blue sealed fracture in 8a showing normalized counts of total CL intensity (black line), 3.2 eV CL intensity (blue line) and Al concentration (grey line). Note that the location of the profile was chosen in a red/orange rather than blue/violet luminescent grain, to clearly see the variation in 3.2 eV emission with respect to the total CL intensity.

The quartz in the quartz-muscovite gouge (u195) is not as homogeneous in CL as the starting material for the pure quartz gouge (u472), and besides red to violet grains, a small amount of originally bright blue quartz is present (see false-colour SEM-CL images in Fig. 7 and RGB composite in Fig. 8a). The quartz in sealed fractures and overgrowths is dark blue and only weakly luminescent with respect to red or bright blue grain cores (compare Fig. 8a–c; Fig. 8g shows the corresponding BSE image). In red to violet grains, the same relation is observed as in the pure quartz gouge described above, between lower red/slightly higher blue CL peak and increased Al. The spatial correlation between increased blue CL emission (Fig. 8d,f,h) and increased Al concentration (Fig. 8e,h) in u195 is not as strong as in the pure quartz gouge, and is sometimes obscured by the strong emission from originally bright blue quartz. The Al-related 3.2 eV peak is not only present in healed fractures and overgrowths, as in the pure quartz sample, but also in some original quartz (see CL spectra in Fig. 6e,f). When comparing spectra from dark blue luminescent quartz in a sealed fracture to reddish original quartz right next to it, the 3.2 eV peak fitted to the spectrum from the healed fracture is stronger than in the spectrum from the original quartz (e.g., CL spectra 1 and 2 in Fig. 6e,f). Furthermore, there is a fairly good visual correlation between Al concentration and 3.2/1.9 eV ratio (compare Fig. 8e,f).

Although the quartz grains in u195 are still quite angular, hardly any porosity is observed and both dissolution structures and precipitated quartz (mostly in sealed fractures and sometimes as overgrowths filling up pore space) are clearly observed (Fig. 7a–d). Generally, dark blue luminescent quartz overgrowths or rims are only observed on quartz-quartz contacts, and not on quartz-muscovite contacts. As described by^4^ the sample shows a boundary-parallel zone of reduced grainsize (roughly the top half of Fig. 7a). Muscovite (black in CL) appears to be partly concentrated in thin bands that are more or less continuous on the scale of the SEM-CL images. This fits with the analyses of Niemeijer^4^, who concluded that a semi-continuous muscovite foliation developed at least partly in this sample. The image in Fig. 7a at full resolution shows that muscovite bands are thinner and more closely spaced in the fine-grained zone towards the top. In between the muscovite bands, the quartz grains appear fractured and resealed with dark blue luminescent quartz, thereby elongating the original grains. This is particularly obvious from the bright blue fragments that clearly stand out in the redder majority of quartz grains.

The CL image taken in the main body of the sample (Fig. 7c) shows opposite orientations of contacts with dissolution structures (red circles) and contacts with dark blue luminescent precipitated quartz (yellow ellipses), with dissolution contacts at an angle of ~ 45°, and precipitation contacts at an angle of ~ 115° to the top surface of the sample. However, these values represent no more than an indication of the structure throughout the samples, as contact geometry was not studied systematically and can be reworked as deformation progressed during the experiment. Figure 7e, for example, shows a grain with a dark blue luminescent overgrowth indenting into another grain. Below the indentation point, in the red indented grain, a thin brighter red luminescent line can be observed. EBSD measurements (inset in top right circle in Fig. 7e) confirm that this is a Dauphiné twin boundary, which are often observed as brighter red lines in CL images of quartz^14^. The geometry of the apparent indentation with the red line around it suggests that a mechanical Dauphiné twin formed as a result of stress at the contact between the grains, extending radially from the indentation point. Also EBSD measurements in other parts of the sample confirm that many Dauphiné twins are present at quartz-quartz contacts throughout the sample.

In some grains, also red luminescent healed fractures were observed (e.g., in the top right corner of Fig. 7c). These are also present in the compacted, but unsheared starting material.

### CL of polishing material

Polishing material (Al_2_O_3_) shows a strong bright blue CL emission which is clearly visible in SEM-CL images and hyperspectral maps of the quartz gouge (u742). This blue emission from remnant polishing material is easily distinguished from blue CL in quartz by the strong 3.6 eV peak in CL spectra from the polishing powder (Fig. 6d). This peak is not present in the blue luminescent precipitated quartz. Furthermore, polishing powder generally has a different shade of blue in SEM-CL images (e.g., bright blue areas between grains in the bottom right of Fig. 2c and in the pores in Fig. 3a) compared to precipitated quartz. The polishing grains stand out clearly in compositional maps by their high aluminium count (black spots in Fig. 5e), and when comparing CL to SE images, blue luminescent quartz is usually easily distinguished from bright blue luminescent polishing powder in pores and cracks (e.g., top right pore in Fig. 3a,b, and right pore in Fig. 3c,d).

## Discussion

In the two experimentally sheared samples described in this study, a clear difference is observed between CL emission of quartz in the starting material and in deformed material. The deformed samples contain blue luminescent quartz in sealed cracks, pores, and grain overgrowths on the originally mostly red to violet luminescent quartz grains. This blue luminescent quartz is not present in the undeformed samples. It is likely that (most of) the blue quartz precipitated during the experiments, and not by dumping of dissolved silica as a result of cooling at the end of the experiments, for two reasons. First, microstructures were observed that can only have formed if the blue luminescent quartz already precipitated during the experiment, then indenting into other grains with ongoing deformation. In Figs. 2c and 3a the white arrows and light grey ellipse respectively indicate locations where blue luminescent quartz indents into original (red luminescent or zoned) quartz grains. In Fig. 7e a Dauphiné twin is observed, extending radially from the point where a dark blue luminescent quartz indents into a red luminescent original grain. Second, quartz precipitation from a saturated fluid at 600 °C to room temperature would result in a porosity reduction of about 0.4%, while the image surface area of blue luminescent (precipitated) quartz in the pure quartz gouge (u742) varies between ~ 2% in the body of the sample to up to ~ 23% in parts of the pulverized zone adjacent to the slip surface, suggesting a much larger volume of quartz has precipitated than would be possible from quenching at the end of the experiment.

Similar Al-rich quartz precipitation and fracture fills precipitated during experiments were observed in CL images of samples from compaction experiments^17^ and precipitation experiments^18^ on quartz. Our results on materials with known starting CL show that variations in CL emission can be useful to trace quartz precipitation also in shear deformation experiments. Especially sealed fractures are usually hardly or not at all visible in SE or BSE images, while they stand out clearly in CL images, which makes CL an excellent microstructural analysis tool that avoids underestimation of the role of fracturing in deformation experiments. Scanned SEM-CL images provide high spatial resolution, but spectral CL analysis in combination with chemical measurements adds useful information about the possible source of CL emission.

## Strain accommodation and deformation mechanisms

In the pure quartz sample (u742), the presence of low angle boundaries < 10° (Figs. 2e and 4c) and fractures that are wider (opening) on one side (Fig. 3c) form evidence for small rotations of quartz fragments. Evidence for larger rotations is lacking, because boundaries resulting from rotations > 10° are, by definition, indistinguishable from regular grain boundaries in EBSD. Evidence for displacement of fragments is lacking, but would be hard to recognize. The observation that fragments with the same zoning pattern are found close to each other (e.g., Fig. 3c) suggests displacements were small. EBSD measurements show that preferred orientation is very weak either away from the slip surface or in the pulverized zone adjacent to it. The pattern of the c-axis maxima might, at first sight, be interpreted to indicate basal slip (c-axis sub-parallel to slip direction) in the top part, and prism slip (c-axis maxima perpendicular to slip direction) in the rest of the sample. On closer inspection, however, while the c-axes superficially fit the characteristic patterns of basal and prism slip^25^, the a-axes are not in the right location. Additionally, we do not consider the MUD value of ~ 1.5 in both parts of the sample as very significant and conclude that any potential contribution of crystal plastic deformation mechanisms to shear must have been very minor. CL images show the presence of precipitated quartz in sealed fractures and grain overgrowths, with more precipitated quartz present near the slip surface than in the body of the sample.

The mechanical behaviour of the experiment, with stick–slip events followed by stable sliding at a low friction coefficient (Fig. 1), and the presence of a pulverized zone next to the slip surface (Figs. 3e–h and 4a), suggests initial localization along a boundary shear, which is typically related to unstable sliding^26^. Since stick-slips occur from the onset of sliding, grain size reduction probably does not play a large role in the initial localization. The first stress drop was 28.3 MPa and was accompanied by an audible acoustic emission. Displacement during the stress drop is estimated to be about 140 µm, based on the apparatus stiffness of 200 MPa/mm. This is sufficient displacement to cause significant local frictional heating, leading to local melting^27,28^. We found clear injection of Ni-rich material into the sample (Fig. 3c,d), indicating that some melting of the piston material (Ni-alloy René-41, with a melting point of T = 1350 °C in the absence of stress) must have occurred. Further stick-slips occur with a decreasing peak friction but with similar or larger stress drops (Fig. 1). This indicates that the slip surface remains localized, which might be promoted by grain size reduction resulting from pulverization during stick–slip pulses, leading to increased compaction and healing by enhanced dissolution and precipitation in the fine-grained zone, thereby strengthening this zone and further favouring localization on the slip surface. Smoothing of the surface might result from removing of asperities by enhanced dissolution and precipitation^3^, and from filling pores and irregularities with fine-grained quartz and smeared out piston material (e.g., Fig. 3e, f), effectively flattening the surface and causing a decrease in the peak friction. Healing (by quartz precipitation) and thus strengthening of the pulverized zone, again favouring localization on the slip surface, might furthermore be enhanced by the high fracture density, since quartz has been found to precipitate preferentially on fracture surfaces rather than grain boundaries^18^. Reduced porosity in this zone could also lead to stronger localization, as Giger et al.^2,17^ argued that below a porosity of 7%, pores can become disconnected, resulting in locally increased pore fluid pressure and reduced frictional resistance.

In the quartz-muscovite mixture (u195), the opposite orientation of contacts with dissolution structures with respect to contacts with dark blue CL quartz (red circles and yellow ellipses respectively in Fig. 7c,d) suggests that at least some precipitation of quartz took place in strain shadows, thereby accommodating part of the shear deformation. This fits with Niemeijer^4^, who concluded that in this particular sample part of the deformation was accommodated in the body of the gouge sample by frictional-viscous flow, a model in which deformation is accommodated by a combination of frictional sliding (here, on the muscovite) and dissolution–precipitation creep (of the quartz grains)^29–31^. The CL images provide additional insights: first that the precipitation also occurs in sealed fractures, so the length scale for dissolution and precipitation is less than the length of the quartz domains. This implies that fracturing might be more important in frictional-viscous flow than generally assumed. Secondly, as discussed by Niemeijer^4^, the foliation-parallel quartz-muscovite bands are not continuous and are interrupted by quartz-quartz indentations, which will act as high friction asperities along the low friction quartz-muscovite interfaces. CL images also nicely show elongation of quartz grains parallel to the slip surface, in the fine-grained boundary zone (top half of Fig. 7a). Bright blue original grains, now fragmented and sealed together by dark blue luminescent quartz, might be used as strain markers in this zone.

EBSD maps in both samples show a few low angle boundaries (< 10°), but these are associated with sealed fractures rather than produced by dislocation activity (e.g., yellow ellipses in Fig. 2c–e). In the body of u742, the microstructure in EBSD can superficially resemble a core-mantle microstructure resulting from dynamic recrystallization (Fig. 2e). However, experimental conditions and other microscopic results (CL, SE, and BSE images) show this is obviously a microstructure resulting from cataclastic and dissolution–precipitation processes. The formation and motion of Dauphiné twins at grain contacts occurred in both samples. Dauphiné twinning does not directly accommodate plastic strain, although the twinning can relax the stresses at the contact, thus reducing the driving force for dissolution. However, there is no obvious relation between Dauphiné twins and location of precipitated blue luminescent quartz.

### Cathodoluminescence

Total intensity CL maps show a lower intensity CL emission from the blue precipitated quartz. In recent studies on recrystallization of deformed quartz, decreased total CL intensity was linked to a reset of the Ti concentration in quartz^19,20^. Also in our samples we observe a decreased total CL intensity in the precipitated quartz with blue CL emission. However, detailed analysis of the CL emission spectra from these sample areas suggests that, although the total CL intensity might be reduced, the blue appearance is caused by the combination of a relatively lower red CL emission, plus a broad blue emission that is slightly stronger in the newly precipitated quartz than in the original material (see the comparison of normalized CL spectra from both original and blue luminescent quartz in sealed fractures in Fig. 6c,g). A lower 1.9 eV (red) emission in the blue luminescent quartz relative to the original quartz suggests the presence of fewer non-bridging oxygen hole centre (NBOHC, ≡Si–O•, a hole in an oxygen orbital) defects or less water (OH-groups) in the newly precipitated blue quartz^32^. Peak fitting to the CL spectra (Fig. 6) suggests that the increased blue emission is the result of slightly stronger emission peaks both at ~ 2.7 eV and ~ 3.2 eV. The 2.7 eV emission can be related to Ti^4+^ incorporation in the quartz crystal structure^12^, or to a structural defect, the self-trapped exciton (STE) associated with E’_1_ centre (≡Si•, an unpaired electron in an Si atom bonded to three oxygen atoms)^32^. If Ti plays a role in the CL emission of the quartz, the increased 2.7 eV peak would imply increased Ti concentration in the precipitated quartz. Although increased temperature near the slip surface, as indicated by molten piston material, might allow for some Ti incorporation during the experiments, no significant variations in Ti concentration were detected between original (red) and precipitated (blue) quartz. There is no obvious source of extra Ti in the sample or the experimental setup, other than the ring shear pistons, which are composed of René-41 metal alloy containing 3–3.3% Ti. Therefore, we expect that Ti concentration in the precipitated quartz is not increased significantly and that the slightly stronger blue 2.7 eV peak is mainly caused by the presence of structural defects (STE), with possibly a minor contribution from Ti derived from the pistons.

The 3.2 eV peak can be related to Al^3+^ substitution of Si in the quartz lattice^9,33^. The contribution of Al^3+^ to the blue CL emission is supported by the observed spatial correlation of blue CL, increased Al concentration, and increased 3.2/1.9 eV ratio (Figs. 5a,e,f and 8a,e,f). Most likely the mullite (aluminium silicon oxide) insulation tubes used in the experimental setup is the main source of the extra Al in the newly precipitated quartz, with a potential minor contribution from the René-41 ring shear pistons, containing 1.4–1.8% Al.

The brighter red to yellow patches that are present in some quartz grains, mostly near the slip surface, in u742 (Figs. 2c, 3e,g and 4a) cannot directly be explained by our data. The presence of the patches near the slip surface and at fractures and crack tips suggests the yellow CL might be stress related. Hamers et al.^14^ showed that subgrain boundaries in quartz, which have a high dislocation density, show increased red CL emission. Future research using Raman spectroscopy, high-angular resolution EBSD or transmission electron microscopy could reveal a potential relationship of the bright red to yellow CL patches with residual stress, strained bonds, incipient (fracture) damage or even high dislocation density due to local crystal plastic deformation induced by heating of the slip surface.

## Conclusions

Cathodoluminescence spectroscopy and false-colour SEM-CL images clearly show that dissolution and precipitation of quartz take place in quartz and mixed quartz-muscovite gouge during low velocity shear experiments under hydrothermal conditions. In both experiments microstructural evidence for dissolution–precipitation processes was found, and blue luminescent quartz associated with increased Al concentration precipitated in sealed fractures and in rims and overgrowths. Indentations of both original and precipitated quartz into other grains are also evident from SEM-CL images. Mechanical data show that in the quartz gouge a period of stick–slip events was followed by stable sliding with a low friction coefficient, with stable sliding occurring on a slip surface. The quartz near the slip surface was pulverized during stick–slip pulses and healed by precipitation of quartz in between stick–slip events, or during stable sliding in the second half of the experiment. Although the microstructures in the quartz gouge indicate that some strain accommodation occurred by precipitation of quartz in strain shadows and especially in sealed fractures (i.e., dissolution–precipitation creep), most deformation was localized on the slip surface. In the quartz-muscovite mixture the deformation was probably accommodated in part by brittle and dissolution–precipitation processes in a boundary-parallel zone of reduced grain size, and in part by frictional-viscous flow in the main body of the sample, in which dissolution–precipitation played an important role. SEM-CL images in the body of the sample show evidence for precipitation of quartz in strain shadows, but mainly in sealed fractures. This suggests that in frictional-viscous flow fracturing could be more important than generally assumed. Our results show that CL imaging and hyperspectral mapping, especially in combination with controlled starting materials and chemical analysis, are a powerful tool in microstructural analysis of experimentally deformed quartz-containing gouges.

## Data Availability

The datasets used and analysed in this study are available from the corresponding author on reasonable request.
